# What influences preneoplastic colorectal lesion recurrence?

**DOI:** 10.18632/oncotarget.13628

**Published:** 2016-11-25

**Authors:** Giulia De Maio, Elisa Zama, Claudia Rengucci, Daniele Calistri

**Affiliations:** ^1^ Biosciences Laboratory, Istituto Scientifico Romagnolo per lo Studio e la Cura dei Tumori (IRST) IRCCS, Meldola (FC), Italy

**Keywords:** recurrence, preneoplastic lesions, suppressor and serrated pathway, microbiota

## Abstract

The hypothesis of the local recurrence of preneoplastic lesions was first put forward in the 1950s. Disease recurrence may result from an inherent imbalance in cell proliferation that promotes carcinogenesis in apparently normal mucosa. Our review sheds light on how early preneoplastic lesions could be used to diagnose relapsed preneoplastic and, developing neoplastic lesions. We focus in detail on the clinical-pathological and molecular features of adenoma subtypes and their role in relapsed adenoma and their development into colorectal carcinoma. Moreover, we include the data available on microbiota and its metabolites and their role in recurrence. We strongly believe that a significant improvement could be achieved in colorectal screening by introducing personalized endoscopic surveillance for polyp-bearing patients on the basis of the presence of molecular markers that are predictive of recurrence.

## INTRODUCTION

Colorectal cancer (CRC) is the major worldwide cause of death and the majority of the cases occur in developed regions. Europe has one of the highest incidences of CRC in the world. Notwithstanding, mortality is lower (9% of the total worldwide) than in less developed regions of the world (52% of the total worldwide) [1]. The low mortality rate has been attributed to the impact of routine screening programs [2], which allow the early identification and surgical removal of preneoplastic lesions in asymptomatic patients. Indeed, the risk of developing CRC is not homogenously distributed, and a small subgroup of patients have a higher incidence of CRC that persists after baseline polypectomy [3, 4]. According to the European Society of Gastrointestinal Endoscopy (ESGE) and American Gastroenterological Association (AGA) guidelines, patients with preneoplastic lesions such as adenomatous polyps are assigned to a risk subgroup on the basis of lesion histology [3]. Specifically, patients with high-grade dysplasia adenomas, >20% villous histology, ≥ 10 mm in size, 3 or more adenomas and serrated polyps ≥ 10 mm in size are considered at high risk and advised to undergo close endoscopic surveillance for 1- 3 years. Conversely, patients with up to 2 tubular adenomas, with < 10 mm, low-grade dysplasia, or serrated polyps < 10 mm and no dysplasia are considered to be at low risk of developing CRC. Both the ESGE and AGA recommend a follow up ranging from 5 to 10 years [3]. Follow-up colonoscopy every 10 years is considered adequate for individuals with hyperplastic polyps (HPs) (Table 1) [5].

**Table 1:** Risk of developing colorectal cancer in adenoma patients

	Recommended surveil lance interval (yr)	Serrated adenomas	Characteristics	Conventional adenomas
**No grade**	10	MVHPs GCHPs MPHPs		
**Low grade**	5	SSAs/Ps	< 1 cm < 3 adenomas Low dysplasia < 20% villous component	TAs
**High grade**	3	TSAs	≥ 1 cm	TSAs
			≥ 3 adenomas	TAs
		SSAs/Ps	High dysplasia	TVAs
			≥ 20% villous component	TVs

The idea of field cancerization to clarify the incidence of multiple primary tumors, local recurrence, abnormal tissue near the cancer and multifocal areas of precancerous change emerged in the 1950s [6]. Initially, CRC field cancerization was founded on histopathological parameters including size, number, localization, histology and grade of dysplasia of lesions detected during the baseline polypectomy. Molecular diagnostic procedures have shown field cancerization is caused by the accumulation of early changes in normal-appearing tissue [7–12]. Despite this, there are still no definitive molecular factors capable of identifying which early colorectal lesions are most likely to relapse. Disease recurrence may, in fact, be due to lesions that have been missed or not radically removed. Moreover, an inherent imbalance in cell proliferation may also promote carcinogenesis in apparently normal mucosa [3].

Sporadic CRC develops gradually over the years as a result of genetic and epigenetic modifications. As it is a heterogeneous disease, the analysis of molecular alterations in cancer precursors could help to discriminate between different types of pathogenesis and clinical behavior. This subtype has been classified on the basis of combinations of genetic markers, *e.g*. microsatellite instability (MSI), CpG island methylator phenotype (CIMP), *BRAF* mutation, and/or somatic *KRAS* mutation [13, 14] and two pathways have been identified. The progression of conventional adenoma to carcinoma is known as the suppressor pathway, while the progression of serrated adenoma to carcinoma sequence is described as the serrated pathway. Although few molecular markers are known to be related to adenoma recurrence, some authors have recently discovered that also microbiota influences adenoma and cancer etiology. However, the way in which the gut microbiota influences adenoma recurrence remains to be clarified [15].

We present an overview of emerging strategies that could help us to understand whether early preneoplastic colorectal lesions are capable of predicting the recurrence of preneoplastic and developing CRC. We shed light on current histopathological and molecular features of preneoplastic colorectal lesions and put forward hypotheses about their involvement in recurrence. Although we are not ready to change our surveillance program based on molecular and microbiota characterization, we believe that the identification of biomarkers and bacteria involved into relapse process has potential for improving the personalized endoscopic surveillance of polyp-bearing patients in a screening program.

### The suppressor pathway

The suppressor pathway, also called the canonical or conventional pathway, is found in 80%-85% of CRCs and is known to follow the Fearon and Vogelstein model [16]. Early lesions detected are conventional adenomas: tubular (TAs) or villous (VAs) or tubular-villous adenomas (TVAs) [16, 17]. Conventional adenomas are usually sited in the ascending colon and rectosigmoid [18]. Villous structures constitute ≥ 80% of VAs and > 20% of TVAs and show different morphologies *e.g*. classic villi (long, slender, finger-like projections), palmate villi (leaf-like, broad, branched projections) and foreshortened villi (isolated, slender projections) [19]. TA is the most common subtype constituting 65-80% of all preneoplastic lesions removed. Histologically, TAs have branched tubular glands and are often pedunculated with fewer atypia than VAs. VAs have long, finger-like projections and represent 5-10% of neoplastic polyps. They are frequently sessile and are more likely to show severe atypia or dysplasia than TAs. About 10 to 25% of polyps are TVAs with cellular features of TAs and VAs [20].

These adenomas are characterized by allelic losses in the adenomatous polyposis coli (*APC*) gene which alters the Wnt–β-catenin pathway, activation of prostaglandin signaling induced by inflammation or mitogen-associated upregulation of cyclooxygenase-2 (*COX-2*), *TP53* mutation and loss of heterozygosity at 18q chromosome [17, 21]. Conventional adenomas with high MSI are linked to Lynch syndrome, whereas *APC* germline mutations are correlated with familial adenomatous polyposis [22].

On the basis of the Fearon and Vogelstein model, the bi-allelic inactivation of *APC* followed by oncogenic *KRAS* mutation, which culminates in the inactivation of *TP53*, are the main molecular alterations involved in the progression from conventional adenoma to cancer [16, 17]. Moreover, cancers that develop through this conventional pathway are generally CIMP-negative and show microsatellite stability (MSS) but chromosomal instability (CIN) [23]. Jass et al. detected *KRAS* mutation in 18% of TAs and 50% of VAs. Moreover, mutations in *KRAS* and *BRAF* appeared to be mutually exclusive in the mitogen-activated protein kinase (MAPK) pathway. In fact, *KRAS* was mutated in 27% of adenomas while only 5% showed a *BRAF* mutation [17]. In agreement with this, Kim et al. reported that about half of the conventional adenomas harbored *KRAS* mutations but none showed *BRAF* mutations [24]. Leggett et al. reported similar results, observing that only a small proportion of conventional adenomas contained *BRAF* mutations [25]. It has also been seen that the loss of O^6^-methylguanine DNA methyltransferase (*MGMT*) gene expression correlates with *KRAS* mutation in small TAs [17]. Furthermore, Whitehall et al. reported that phosphatidylinositol-4,5-bisphosphate 3-kinase catalytic subunit alpha (*PIK3CA*) gene mutations were exclusively observed in TVAs [26].

**Figure 1:**
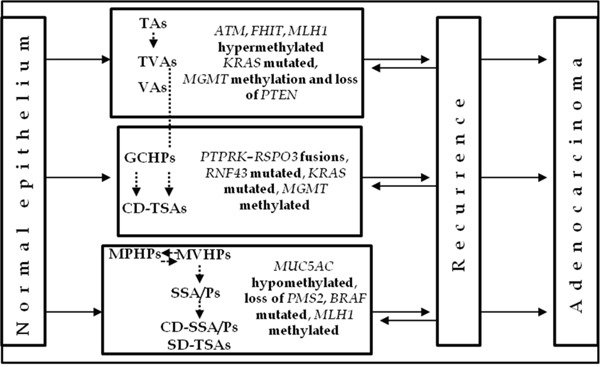
Distribution of lesions according to molecular alterations involved in the etiology and/or recurrence of preneoplastic lesions Schematic diagram of CRC progression and recurrence. Two pathways have been recognized, the suppressor and the serrated pathway. Both sequences involve the progression of normal colonocytes into early and advanced adenomas, with subsequent transformation into early and advanced cancer. Relapsed suppressor and serrated preneoplastic lesions retain the same histological subtype. The boxes show the typical molecular biomarkers for each lesion. The dotted arrows represent the potential connections between adenomas and polyps during their development.

In studies on relapsed colorectal lesions, the promoters of the tumor suppressor genes mutL homolog 1 (*MLH1)*, ataxia telangiectasia mutated *(ATM)* and fragile histidine triad *(FHIT)* were found to be significantly hypermethylated in recurring conventional adenomas [27], while the absence of phosphatase and tensin homolog *(PTEN)* expression correlated positively with local recurrence [28]. It has also been seen that conventional adenomas recur more frequently in patients with conventional adenomas at baseline polypectomy [29] (Figure 1).

### The serrated pathway

Jass and Smith first described the serrated pathway in colorectal carcinogenesis more than twenty years ago [30]. In the literature, two terms are synonymous for serrated lesions, *i.e*. serrated polyps or serrated adenomas [31]. The “serrated pathway” is present in about 15%-20% of sporadic CRCs, the morphological features being the serrated or “sawtooth-like” appearance of the crypts. This feature is considered to be a consequence of cell growth in combination with the delayed migration or failure of cell apoptosis, which leads to an accumulation of epithelial cells. It is now acknowledged that several types of serrated polyps exist and can develop into a subset of invasive tumors via the serrated pathway [32]. According to 2010 WHO guidelines, this pathway has been recognized in sessile serrated lesions *i.e*. HPs, traditional serrated adenomas (TSAs) and sessile serrated adenomas/polyps (SSAs/Ps) [33]. However, a definitive classification for these polyps is still lacking and histological interpretation often differs among pathologists [34–36].

HPs, which represent more than 75% of serrated polyps, are flat or sessile, pale in color, generally < 5 mm, and are usually located in the distal colon. They are the only subtype of polyps with no dysplastic progression. HPs are often larger than other serrated lesions and difficult to visualize endoscopically. Although they are found more frequently than conventional adenomas in younger individuals, their incidence does not substantially increase after the age of 50 [37]. HPs can be divided into microvesicular (MVHPs), goblet cell (GCHPs) and mucin-poor polyps (MPHPs). MVHPs are frequently CIMP-high with *BRAF* mutation. Although they are mainly detected in the descending colon, 10-15% occur in the transverse and ascending colon. Multiple MVHPs are frequent in the rectum. GCHPs often have *KRAS* mutation, are more commonly found in the descending colon, and are usually small [38]. MPHPs have little to no cytoplasmic mucin and show a luminal serration pattern with increased nuclear atypia comprising large, round, hyperchromatic nuclei without pseudostratification. Observational studies report that HPs are not connected with advanced adenomas [39, 40]. Indeed, the co-existence of HPs with adenomas at index colonoscopy does not increase the risk of further adenomas or advanced adenomas at surveillance [41, 42].

In Western populations only 5% of serrated polyps are TSAs. Generally found in the descending colon and with a higher incidence in the elderly [23, 43], they are protuberant or pedunculated lesions similar to conventional adenomas, with an architecture characterized by ectopic crypts at their base. This morphological feature is used to distinguish them from SSAs. TSAs show copious eosinophilic cytoplasm and elongated, outlined nuclei. They can develop conventional or serrated dysplasia, both types of which are believed to be markers of progression to carcinoma [23]. A subset of TSAs with conventional dysplasia (CD-TSAs) is characterized by *KRAS* mutations and silencing of *MGMT* by promoter hypermethylation, CIMP-low phenotype and MSS [44]. Conversely, TSAs with serrated dysplasia (SD-TSAs) are characterized by CIMP-high, *BRAF* mutation and MSS [23].

Numerous studies have shown that individuals with sessile serrated lesions at baseline tend to develop additional sessile serrated lesions over time [29, 45, 46]. Of note, DNA mismatch repair (MMR) proteins are intact in all cases of TSAs, including those with dysplasia [47].

SSAs/Ps without dysplasia are the most clinically important serrated polyps due to their high incidence. These flat or slightly elevated lesions are often located in the proximal colon and generally measure > 5 mm [23]. Histologically, SSAs differ from HPs in their manifestation of atypical structural characteristics due to abnormal proliferation. The HP proliferation zone is situated at the bottom of the crypts. However, in SSAs/Ps, crypt proliferation leads to an increase in asymmetry, *e.g*. T-shaped or inverted L-shaped structures. SSAs/Ps may also have a yellow mucous cap, which makes them easier to identify endoscopically [23, 37]. From a molecular point of view, SSAs/Ps show a high frequency of *BRAF* mutation, CIMP phenotype and MSS [48, 49].

It has been reported that 19% to 27% of CRCs occur at the same site of the primary polypectomy, 18% of patients with large sessile polyps showing residual adenomatous tissue when re-examined [49–53].

Cytological dysplasia in SSAs/Ps (CD-SSAs/Ps) resembles that of conventional adenomas characterized by elongated pencillate nuclei with hyperchromasia, nuclear pseudostratification and amphophilic cytoplasm [23]. The transition between cytological dysplastic epithelium and non-dysplastic SSA/P epithelium may be abrupt, appearing as a ‘collision’ between two lesions and leading to their classification as “mixed hyperplastic adenomatous polyps” or “mixed SSA/P-TA” [54, 55]. CD-SSAs/Ps are characterized by wild type *KRAS*, mutated *BRAF*, CIMP-high status, methylated *MLH1* and MSI [56].

Lazarus et al. reported that recurrence rates of serrated adenoma were higher than those of HPs or conventional adenomas [29]. In a retrospective study, Lu et al. reported that 15% of SSP patients developed cancer or adenomatous polyps with high-grade dysplasia compared to 6% of control patients with HPs or adenomatous polyps at baseline [40]. However, patients with non-dysplastic SSAs/Ps did not develop advanced neoplasia, even though the size and location of the lesions indicated that there was an increased risk of CRC [61, 62]. Moreover, the detection of serrated polyps was indicative of the subsequent development of mature serrated polyps rather than conventional adenomas [29, 57]. Indeed, 94% of patients with serrated adenomas at the first check-up later developed serrated polyps. Furthermore, Teriaky et al. observed new SSPs within the first 3 years of follow-up in patients with SSPs at baseline [45]. Renaud et al. observed that mucin 5AC (*MUC5AC*) gene hypomethylation was an early event in the serrated neoplasia pathway and specifically detected MVHP and SSA/P lesions [58]. Individuals with co-existing SSAs/Ps and conventional adenomas appear to be a high-risk group [59]. Sekine et al. showed that TSAs commonly harbor genetic alterations leading to the activation of the WNT pathway [60]. In particular, they found that TSA genetic features were *PTPRK-RSPO3* fusions and *RNF43* mutations (Figure 1) [60].

### Interactions between preneoplastic lesions

As previously mentioned, various models of CRC development, starting from preneoplastic lesions, have been hypothesized, each of which is based on different molecular mechanisms. The suppressor and serrated pathways are the best characterized and most clearly distinguishable. However, to date relatively few genetic or epigenetic factors have been correlated with recurrent lesions (Figure 1). TSAs are a heterogeneous group that can be divided into CD-TSAs or SD-TSAs on the basis of specific molecular markers [47, 63]. SD-TSAs have been described as exhibiting areas with features of HPs and/or SSAs/Ps, and the hypothesis that HPs and/or SSAs/Ps may be precursor lesions to TSAs has been put forward [23]. Tsai et al. reported that TSAs with this type of dysplasia develop into in smaller, less aggressive neoplastic lesions than other preneoplastic subtypes [47]. Conversely, CD-TSAs characterized by *KRAS* mutations, *MGMT* hypermethylation, CIMP-low phenotype and MSS, may originate from TVAs and are associated with a poorer prognosis. This pathway is known as the alternative pathway [23, 64]. It has been suggested that TAs may also evolve along this alternative pathway, bypassing TVAs [23, 65]. It is also likely that MVHPs evolve into SSAs/Ps, especially when they are located in the proximal colon [66]. There is ample evidence that MVHPs and SSAs/Ps without dysplasia show overlapping molecular patterns when tested for CIMP, *MLH1, KRAS* and *BRAF* status, which is consistent with the notion that MVHPs are immediate precursors of SSAs/Ps [23]. The methylation silencing of *MLH1*, which leads to the MSI phenotype, is detected as variably decreased *MLH1* expression in dysplastic areas of CD-SSAs/Ps. Of note, although *MLH1* methylation is detectable early in SSAs/P growth, only a reduction or loss of gene expression by extensive methylation is associated with dysplasia and heralds the committed progression of the serrated cancer pathway to malignancy. In lesions with loss of *MLH1*, the *PMS2* gene, one partner in the stabilizing of MutL heterodimer MMR, is also lost [23]. It has also been suggested that MPHPs are MVHPs that have been modified by inflammation and reactive epithelial changes [38]. However, the only molecular marker associated with MPHPs to date is the CIMP-high phenotype. Successor lesions of GCHPs are seldom observed and it is debatable whether they are self-limiting or progress to advanced *KRAS*-mutated serrated polyps, *i.e*. CD-TSAs (Table 2) [66–68].

**Table 2:** Main molecular alterations of all preneoplastic colorectal lesions

	Preneoplastic lesions	*APC*	*KRAS*	*BRAF*	CIMP	Microsatellite	CIN	*MGMT*
**Serrated adenomas**	**MVHPs**			Mutated	High	Stable		
	**GCHPs**		Mutated		Low	Stable		
	**MPHPs**				High			
	**SD-TSAs**			Mutated	High	Stable		
	**CD-TSAs**		Mutated		Low	Stable		Hypermethylated
	**SSAs/Ps**			Mutated	High	Instable		
	**CD-SSAs/Ps**			Mutated	High	Instable		
**Conventional adenomas**	**TAs**	Mutated	Mutated		Low	Stable	+	
	**TVAs**	Mutated	Mutated		Low	Stable	+	Hypermethylated
	**VAs**	Mutated	Mutated		Low	Stable	+	

### Gut microbiota

The microbial communities of the gastrointestinal tract can be modified by diet and environmental factors [69]. Several studies have analyzed the promoting or protecting role of microbial dysbiosis in the etiology of colorectal adenoma-to-carcinoma sequence [70–73], but few have focused on the correlation between the microbiota and colorectal adenoma recurrence. Bacterial species implicated in the carcinogenesis process are *Streptococcus gallolyticus*, *Enterococcus faecalis*, *Bacteroides fragilis*, *Escherichia coli*, *Fusobacterium nucleatum* and *Faecalibacterium prausnitzii* [74, 75]. Bacteria express their pathogenicity through chronic inflammation, DNA damage, and the production of bioactive carcinogenic metabolites. A higher proportion of the phylum *Proteobacteria*, of the genera *Pseudomonas, Helicobacter* and *Acinetobacter* and a lower abundance of the phylum *Bacteroidetes* have been reported in a number of studies focusing on adenoma [76, 77]. An overabundance of the genera *Fusobacterium* and *Prevotella* in tumor samples has been observed by authors assessing differences in the microbiota between tumors and matching normal colon tissue [78–82]. These results indicate that adenoma development is potentially related to gut microbiota modifications [15]. In particular, inflammatory processes in the gut microbiota appear to be involved in CRC progression [15–83]. In the study by Dulal et al., the transfer of fecal microbiota from tumor-bearing mice to germ-free mice promoted tumorigenesis in the recipient animals [15]. The microbiota of the latter showed an abundance of the genera *Akkermansia, Odoribacter* and *Bacteroides* similar to that of the donor mice. The authors also reported that gut microbiota can be manipulated with antibiotics or probiotics to inhibit adenomas and CRC progression [15].

It is known that colonic microbiota affects a broad range of metabolic processes leading to favorable or detrimental effects. Although metabolites produced by microbiota are thought to play a role in CRC progression, little is known about the function of the majority of gut bacteria and their metabolites. Zackular et al. reported that subjects with adenomas and CRC showed a loss of the genera *Clostridium* and *Bacteroides* and of the family *Lachnospiraceae* in their colonic microbiota [83]. Each of these protective bacteria is a producer of short chain fatty acids (SCFAs) in the colon. SCFAs are metabolites that supply nutrients to colonocytes, thus maintaining the epithelial homeostasis. Specifically, the SCFA butyrate has been shown to have anti-tumorigenic features, including inhibition of tumor cell proliferation, initiation of apoptosis in tumor cells and mediation of T-regulatory cell homeostasis. The depletion of these protective bacterial populations, associated with an improvement in pathogenic populations, probably acts as a tumorigenic stimulator [83].

Nugent et al. demonstrated that 23 metabolites contribute to the development of adenoma, mainly as a result of an increase in prostaglandin E2, an inflammatory metabolite, and a decrease in 5-oxoproline and diketogulonic acid, two antioxidant-related metabolites [84]. Pathway studies have shown that numerous metabolites are significantly related to inflammatory response, carbohydrate metabolism and gastrointestinal diseases. An abundance of the genus *Bifidobacterium* has also been found in patients with adenomas. These metabolites were differently correlated with the bacteria of healthy and sick individuals, suggesting that bacteria metabolic products may be responsible for adenoma and CRC development.

Ito et al. detected the species *Fusobacterium nucleatum* bacteria in HPs, SSAs, TSAs and non-serrated adenomas but found that the presence of this bacterium was not significantly associated with lesion histology [85]. Positivity was, however, significantly correlated with CIMP-high status and larger tumor size, suggesting that *Fusobacterium nucleatum* plays a role in the initial phase of CRC tumorigenesis [85–86].

CRC bacterial dysbiosis is associated with a decreased abundance of obligate anaerobes, an increase in potentially pathogenic bacteria, and a reduction in the proportion of beneficial butyrate-producing bacteria, implying that microbial metabolites are involved in CRC etiology [87–89]. Furthermore, some dietary elements are metabolized by symbiotic microbiota into bioactive food components believed to prevent cancer [69]. The way in which gut microbiota and specific bacteria influence adenoma and CRC development has yet to be clarified. Further research is warranted to determine a correlation between the microbiota, metabolome and preneoplastic/neoplastic lesions.

## CONCLUSIONS

The current review provides an overview of the state-of-art of research into field cancerization and recurrent lesions in colorectal tissue. By examining the histopathological and molecular features of colorectal preneoplastic lesion subtypes, we aimed to show that early preneoplastic colorectal lesions can be used to identify individuals at risk of recurrent preneoplastic lesions and of developing CRC. The active surveillance of subjects with preneoplastic lesions represents an important step forward in preventing CRC and in reducing mortality [90]. It is believed that genetic and environmental factors determine the predominant type of recurrent polyps after baseline polypectomy [29]. In fact, similarities have been found between the type of lesion found at baseline colonoscopy and that detected during follow-up [29, 45]. However, the majority of studies conducted to date focus on the difference between CRC and healthy tissue, only a few evaluating the different precursors of CRC and even fewer assessing the role of recurrent preneoplastic lesions in the development of CRC.

According to the top-down model, genetic modifications occur in healthy tissue of the upper crypt compartment of early conventional lesions [91]. Conversely, genetic and/or epigenetic modifications of serrated pathway early lesions originate from the lower crypt compartment but have not yet been fully characterized. There is evidence to support that an epigenetic program regulated by polycomb repressive complexes maintains the specific functions of the lower compartment [64]. An “epigenetic memory” in lower crypt cells may be present in early preneoplastic lesions and may predispose to the development of neoplastic disease. This may explain why precursor lesions proliferate downwards or laterally, are age-related, rapidly progress, and are more likely to have a CIMP phenotype.

Generally, it has been seen that conventional adenomas follow the traditional pathway of developing cancer with CIN, CIMP-negative, MSS, *BRAF* wild type and *KRAS* mutated. Conversely, principals precursors lesions of the serrated pathway are characterized by *BRAF* mutation, CIMP-high and MSS phenotype.

In recent years, the microRNA signatures of conventional, serrated preneoplastic, and neoplastic lesions have been extensively studied [92, 93, 94, 95]. Some miRNA shed new light into the pathogenic mechanisms underlying adenoma-to-carcinoma progression [96]. However, no clear molecular signature exists for all of the preneoplastic colorectal lesions identified to date, a number of markers would seem to play a role in more than one lesion.

For this reason, a molecular characterization of preneoplastic lesions would help to better classify preneoplastic lesion subtypes, in particular those with a higher risk of recurrence and with worse histopathological features. It would also facilitate planning of surveillance measures for polyp-bearing patients. Furthermore, a better understanding of microbiota found in relapsed lesions could shed light on the bacteria present in preneoplastic and neoplastic colorectal lesions. Such knowledge could be implemented in CRC screening programs to improve the effectiveness of personalized endoscopic surveillance programs for polyp-bearing patients.
